# Five new species and one new record of armored spiders (Araneae, Tetrablemmidae) from Thailand

**DOI:** 10.3897/zookeys.1279.189587

**Published:** 2026-05-12

**Authors:** Songlu Shi, Dongju Bian, Yanfeng Tong, Shuqiang Li

**Affiliations:** 1 College of Life Science, Shenyang Normal University, Shenyang 110034, Liaoning, China Institute of Applied Ecology, Chinese Academy of Sciences Shenyang China; 2 CAS Key Laboratory of Forest Ecology and Silviculture, Institute of Applied Ecology, Chinese Academy of Sciences, Shenyang, 110016, China College of Life Science, Shenyang Normal University Shenyang China; 3 College of Life Sciences, Anhui Normal University, Wuhu, Anhui 241000, China College of Life Sciences, Anhui Normal University Wuhu China

**Keywords:** *
Ablemma
*, diagnosis, distribution_,_ new species, *
Shearella
*, taxonomy, *
Tetrablemma
*

## Abstract

Five new cave-dwelling species and one newly recorded litter-dwelling species belonging to the family Tetrablemmidae are described from Thailand, i.e., *Ablemma erna* Lehtinen, 1981 (♂♀), *A. theppratan* Tong & Li, **sp. nov**. (♂♀), *A. yamae* Tong & Li, **sp. nov**. (♂♀), *Shearella khaoplu* Tong & Li, **sp. nov**. (♂), *S. thamphothisat* Tong & Li, **sp. nov**. (♂) and *Tetrablemma lorkor* Tong & Li, **sp. nov**. (♂). Diagnoses and illustrations for all six species are given.

## Introduction

Tetrablemmidae O. Pickard-Cambridge, 1873, also known as armored spiders (Lehtinen 1981; Martínez 2025), currently comprises 153 extant species placed in 27 genera (WSC 2026). These spiders are predominantly distributed across tropical and subtropical regions, where they occupy microhabitats such as leaf litter, soil, and caves (Lehtinen 1981; Burger 2005). Tetrablemmids are very small (0.7–3 mm), cryptic haplogyne spiders. They are readily distinguished from other spider groups (except Pacullidae Simon, 1894) by their parallel, strap-like lateral abdominal sclerites. From Pacullidae, they differ in their considerably smaller size (body length less than 3 mm), smooth cuticle, and the presence of a pair of large membranous receptacles in the endogyne (Shear 1978; Lehtinen 1981; Jocqué and Dippenaar-Schoeman 2006).

Tetrablemmids are very diverse in Southeast Asia, 57 extant species belonging to 13 genera have been described from this region (WSC 2026): *Ablemma* Roewer, 1963 (20 species), *Bacillemma* Deeleman-Reinhold, 1993 (1), *Borneomma* Deeleman-Reinhold, 1980 (2), *Brignoliella* Shear, 1978 (12), *Fallablemma* Shear, 1978 (1), *Indicoblemma* Bourne, 1980 (2), *Lehtinenia* Tong & Li, 2008 (1), *Maijana* Lehtinen, 1981 (1), *Pahanga* Shear, 1979 (3), *Singalangia* Lehtinen, 1981 (1), *Singaporemma* Shear, 1978 (5), *Sulaimania* Lehtinen, 1981 (2) and *Tetrablemma* O. Pickard-Cambridge, 1873 (6).

In Thailand, the spider family Tetrablemmidae remains poorly documented. To date, a total of five species belonging to four genera have been recorded: *Ablemma ruohomaekii* Lehtinen, 1981, *Bacillemma leclerci* Deeleman-Reinhold, 1993, *Indicoblemma lannaianum* Burger, 2005, *I. monticola* (Lehtinen, 1981) and *Singaporemma takense* Yan & Lin, 2018 (Lehtinen 1981; Deeleman-Reinhold 1993; Burger 2005; Yan and Lin 2018).

In this paper, five new cave-dwelling species are described from Thailand. Furthermore, a leaf-litter-inhabiting species, *Ablemma erna* Lehtinen, 1981, is recorded for the first time from Thailand.

## Materials and methods

The cave-dwelling individuals were collected by hand, and the litter-dwelling individuals were collected by sifting leaf litter. The specimens were examined using a Leica M205 C stereomicroscope. Details of body parts and measurements were studied under an Olympus BX51 compound microscope. Male and female internal genitalia were mounted on excavated slides with clove oil and subsequently examined. Photos were taken with a Canon EOS 750D zoom digital camera (18 megapixels) mounted on an Olympus BX51 compound microscope and assembled using Helicon Focus v. 3.10.3 image-stacking software (Khmelik et al. 2005). All measurements in the text are expressed in millimetres. The distribution map was generated with ArcGIS v. 10.2 (ESRI Inc.). All materials studied are deposited in the Shenyang Normal University (SYNU) in Shenyang, China.

Terminology mainly follows Lehtinen (1981) and Tong and Li (2008). The following abbreviations are used in the figures and text: **ALE** = anterior lateral eyes; **AS** = anal scutum; **AT** = anterodistal tooth; **BP** = basal process; **CL** = cheliceral lamina; **Csp** = crescent-shaped protrusion; **DP** = distal process; **Em** = embolus; **EP** = epigynal pit; **IVP** = inner vulval plate; **Pa** = preanal scutum; **Plc** = posterolateral corners; **PLE** = posterior lateral eyes; **PME** = posterior median eyes; **Pog** = postgenital scutum; **Pu** = pulmonary scutum; **Spd** = sperm duct; **SR** = seminal receptacle; **ST** = small tubercle; **TP** = triangular process; **VD** = vulval duct; **VS** = vulval stem.

## Taxonomy

### Family Tetrablemmidae O. Pickard-Cambridge, 1873

**Subfamily Ablemminae Lehtinen, 1981**

#### Genus Ablemma

Taxon classification

Animalia

AraneaeTetrablemmidae

Roewer, 1963

19EF0BD0-5CA3-5AF8-BAFC-E0AE1BA8DE40

##### Type species.

*Ablemma baso* Roewer, 1963 from Indonesia, Su­matra.

##### Diagnosis.

See Lehtinen (1981).

##### Composition.

Thirty species, including two described here in two subgenera *Ablemma* and *Bannia* Lehtinen, 1981.

##### Distribution.

Borneo (7 species), Caroline Islands (1), China (2), Indonesia (7), Japan (1), Malaysia (2), New Guinea (5), Philip­pines (2), Singapore (1), Solomon Islands (1) and Thailand (4).

#### Ablemma (Bannia) erna

Taxon classification

Animalia

AraneaeTetrablemmidae

Lehtinen, 1981

CC95EAEF-1D52-52C9-A269-07B9669679CE

Figs 1, 2, 3, 4

Ablemma (Bannia) erna Lehtinen, 1981: 49, figs 154, 171–172, 177, 189.

##### Material examined.

Thailand • 4♂5♀ (SYNU-F-5801–5809); Satun, Thung wa Dist., evergreen forest, bottom of cliff, litter sifting; 7°06'16.51"N, 99°47'30.25"E, 26 m elev.; 30.XI.2013; F. Ballarin leg. • 3♂9♀ (SYNU-F-5967–5978); 7.XII.2013; other data same as above; • 1♀ (SYNU-F-5858); Nakhon Ratchasima, Sakarat environmental research forest, semi-evergreen forest near lake shore, litter sifting; 14°29'49.44"N, 101°54'56.52"E, 478 m elev.; 25.XI.2013; F. Ballarin leg.

**Figure 1.**
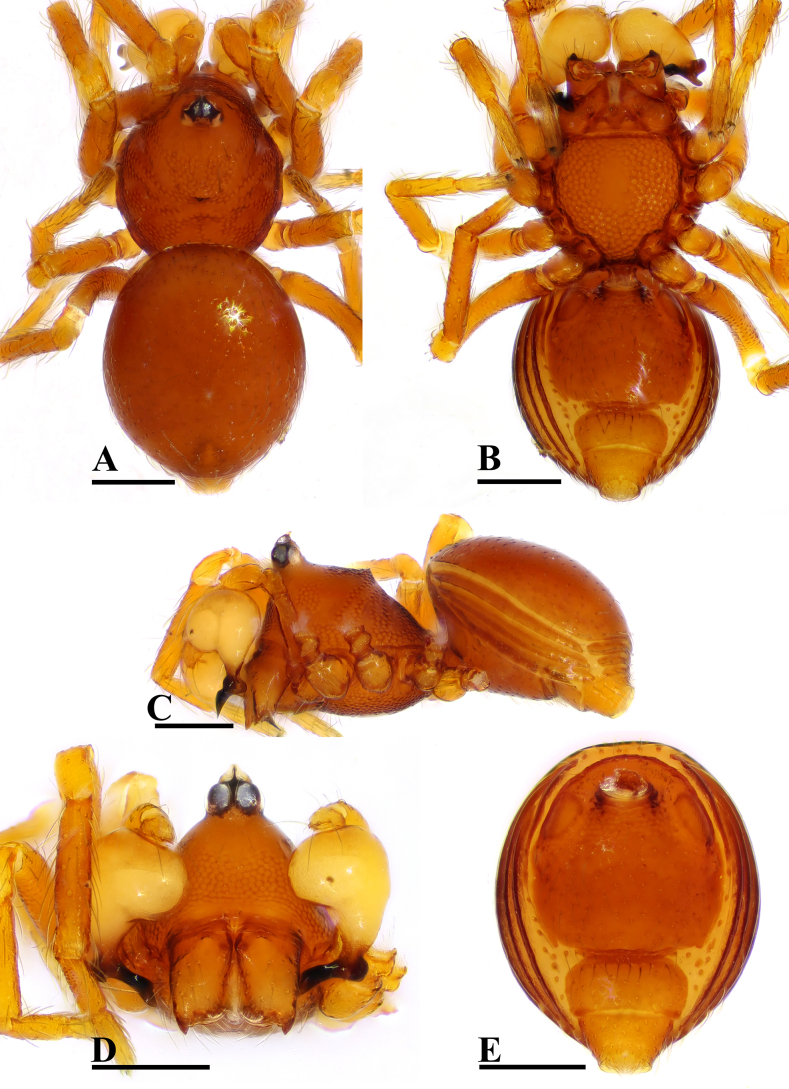
*Ablemma erna* Lehtinen, 1981, male. **A**. Habitus, dorsal view; **B**. Habitus, ventral view; **C**. Habitus, lateral view; **D**. Prosoma, anterior view; **E**. Abdomen, ventral view. Scale bars: 0.2 mm.

**Figure 2.**
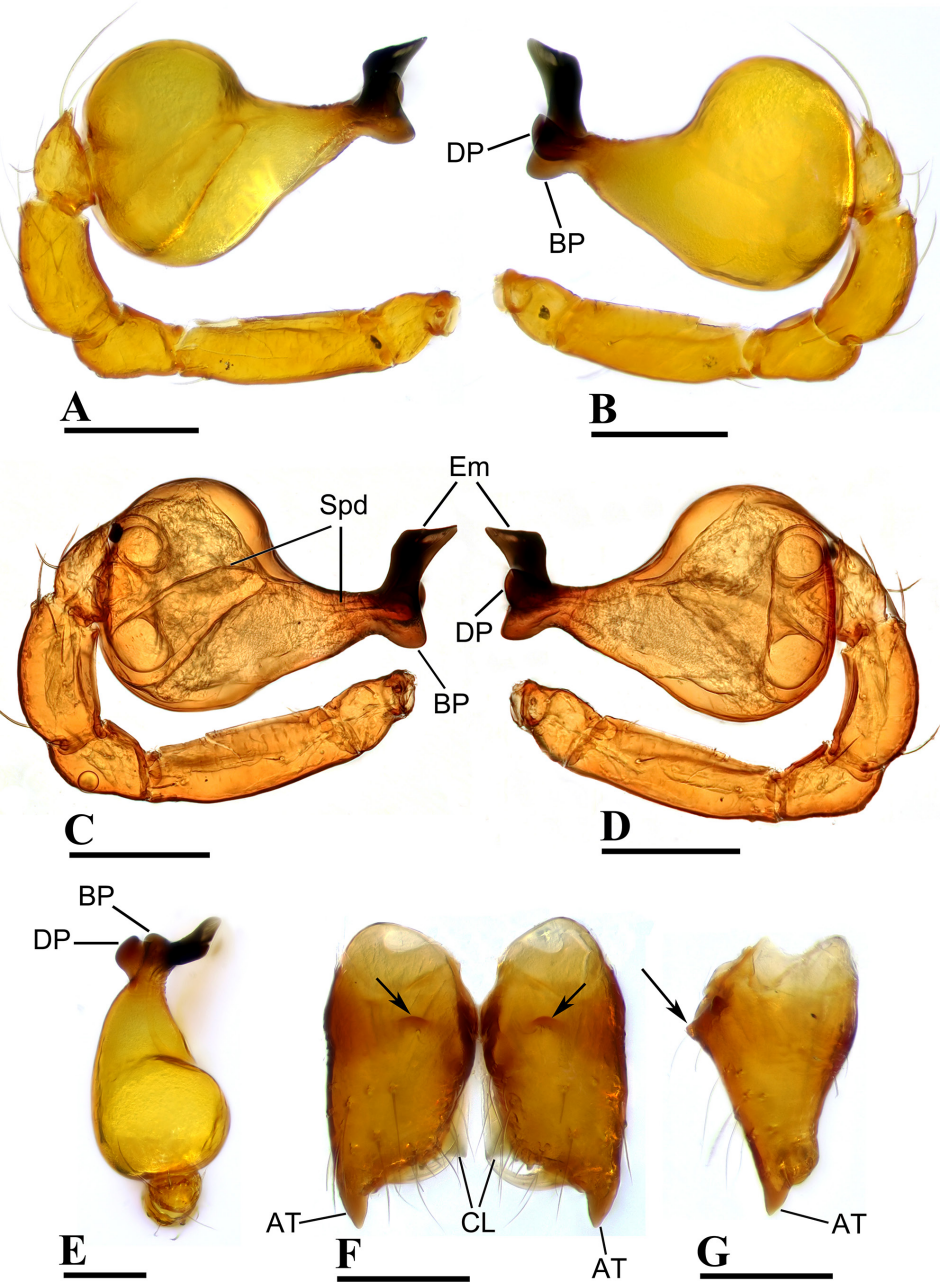
*Ablemma erna* Lehtinen, 1981, male. **A, C**. Left palp, prolateral view; **B, D**. Left palp, retrolateral view; **E**. Left palp, dorsal view; **F**. Chelicerae, anterior view; **G**. Chelicerae, lateral view; arrows show outgrowths in (**F**) and (**G**). Abbreviations: AT = anterodistal tooth, BP = basal process, CL = cheliceral lamina, DP = distal process, Em = embolus, Spd = sperm duct. Scale bars: 0.1 mm.

**Figure 3.**
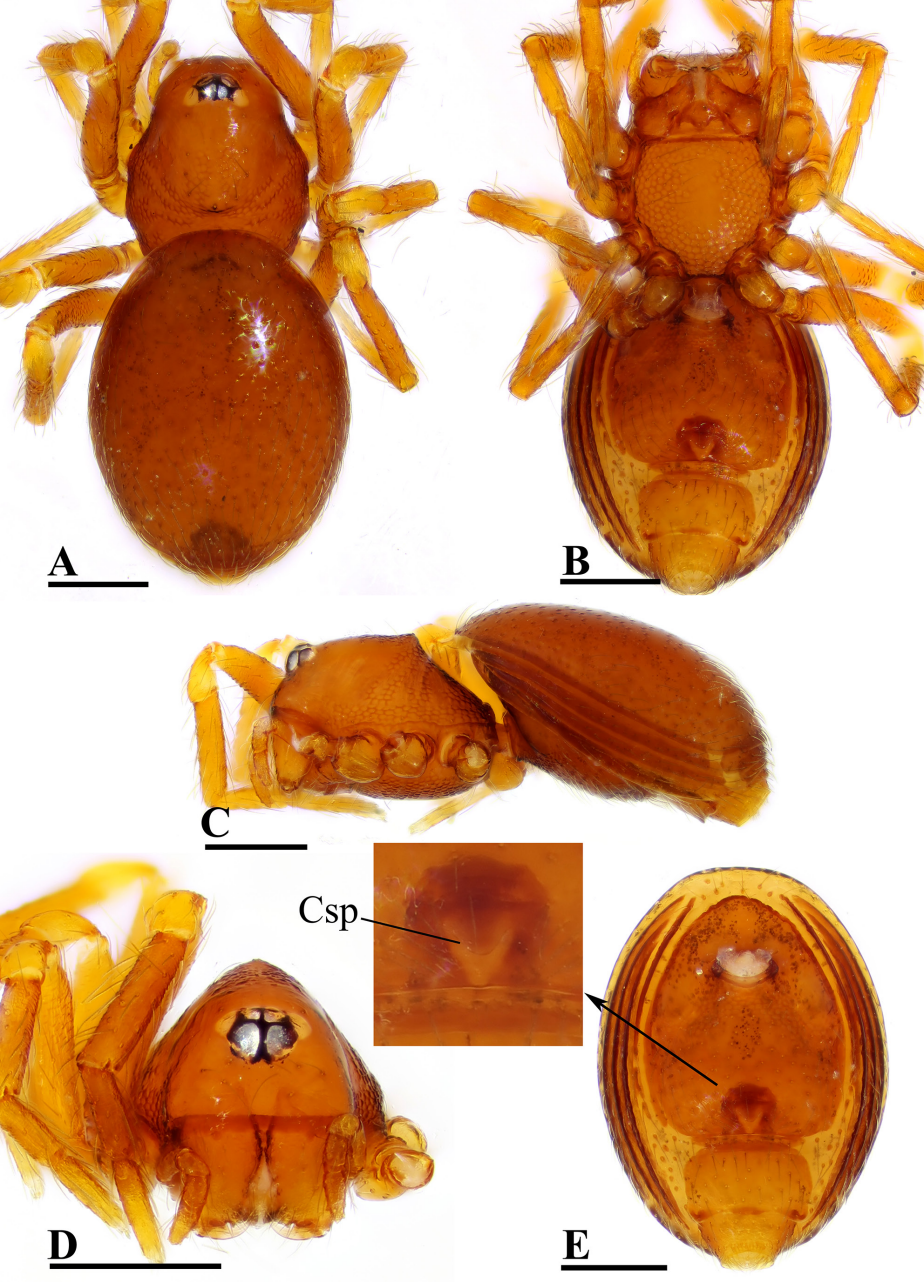
*Ablemma erna* Lehtinen, 1981, female. **A**. Habitus, dorsal view; **B**. Habitus, ventral view; **C**. Habitus, lateral view; **D**. Prosoma, anterior view; **E**. Abdomen, ventral view, arrow shows detail of genital area. Abbreviations: Csp = crescent-shaped protrusion. Scale bars: 0.2 mm.

**Figure 4.**
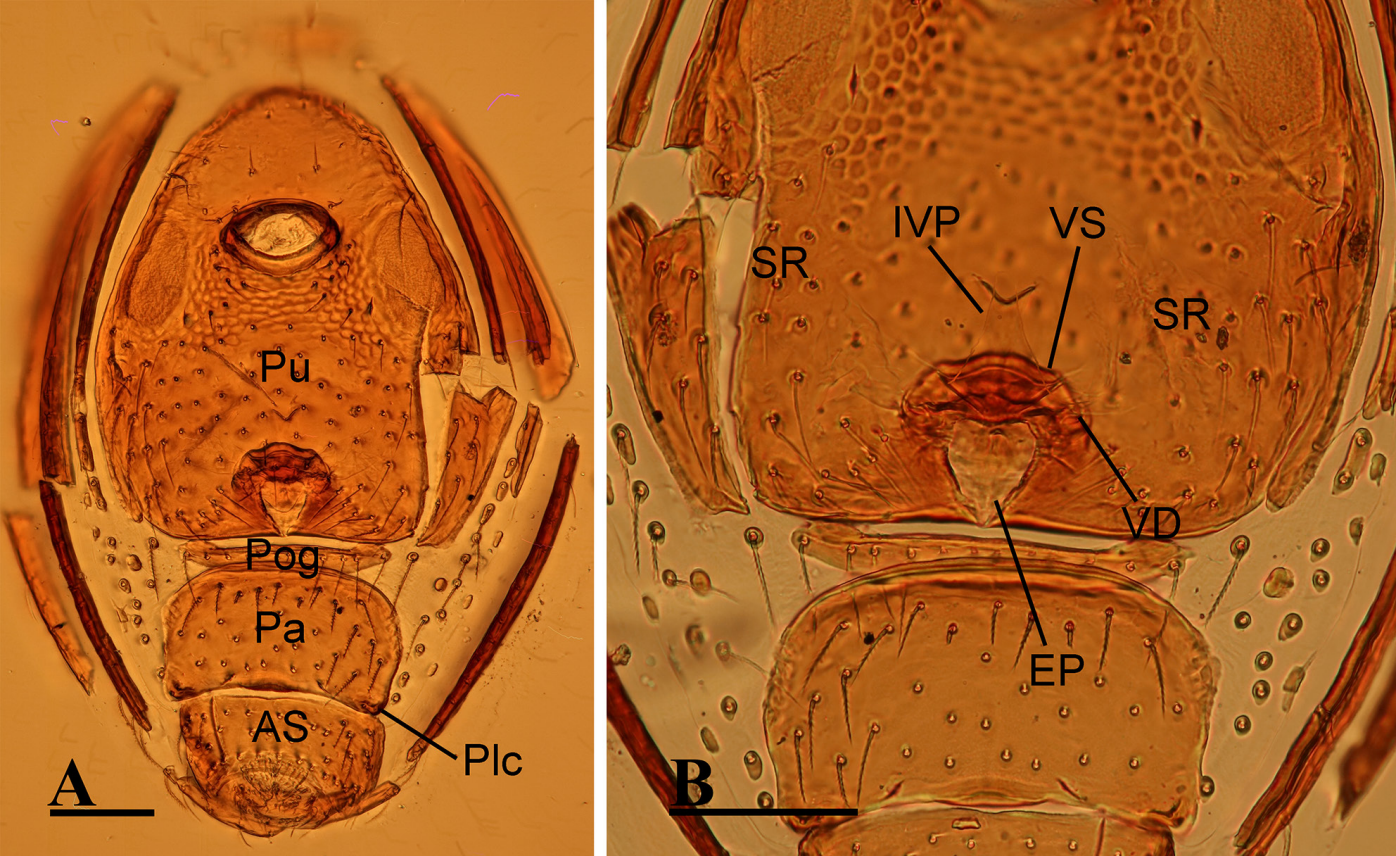
*Ablemma erna* Lehtinen, 1981, female. **A**. Abdomen, ventral view; **B**. Endogyne, dorsal view. Abbreviations: AS = anal scutum, EP = epigynal pit, IVP = inner vulval plate, Pa = preanal scutum, Plc = posterolateral corners, Pog = postgenital scutum, Pu = pulmonary scutum, SR = seminal receptacle, VD = vulval duct, VS = vulval stem. Scale bars: 0.1 mm.

##### Diagnosis.

This species is similar to *Ablemma kaindi* Lehtinen, 1981 in the flat, falciform embolus, but can be distinguished by the flat carapace (vs. with rounded ridges; cf. Fig. 1C and Lehtinen 1981: fig. 149), the distal process (DP) of bulb (vs lacking; cf. Fig. 2B, D, E and Lehtinen 1981: fig. 164) and the crescent-shaped protrusion (Csp) of endogyne (vs lacking; cf. Fig. 3E and Lehtinen 1981: fig. 180).

##### Description.

**Male** (SYNU-F-5801). Coloration: body reddish-brown; legs yellowish brown. Measurements: total length 1.08; carapace 0.48 long, 0.40 wide, 0.33 high; abdomen 0.63 long, 0.50 wide, 0.36 high; clypeus 0.20 high; sternum 0.31 long, 0.31 wide. Length of legs: I 1.09 (0.34, 0.12, 0.29, 0.17, 0.17); II 1.01 (0.31, 0.12, 0.24, 0.16, 0.18); III 0.91 (0.28, 0.11, 0.20, 0.17, 0.15); IV 1.15 (0.36, 0.12, 0.30, 0.19, 0.18).

***Carapace*** (Fig. 1A, C, D): finely reticulated; 6 eyes, ALE largest, PME smallest, raised on short tubercle; clypeus sloping forward, marginally rounded; cephalic part raised, sloping posteriorly; chelicerae robust, with small conical outgrowth (arrows in Fig. 2F, G) and large anterodistal tooth (AT), cheliceral lamina (CL) well developed; labium triangular, blunt distally; sternum reticulated except medially.

***Abdomen*** (Fig. 1A–C, E): dorsal scutum smooth, covered with sparse setae; pulmonary scutum (Pu) smooth, reticulated on pedicel region, with oval book-lung covers; preanal scutum (Pa) rectangular, ca. 5× longer than postgenital scutum (Pog).

***Palp*** (Fig. 2A–E): femur ca. 2.5× longer than patella, 0.7× shorter than bulb; tibia not swollen, ca. 0.7× of femur length; cymbium small, cup-shaped; bulb obtusely triangular, gradually narrowing, with distal process (DP); sperm duct (Spd) broad basally, tapering gradually; embolus (Em) flat, falciform, with basal process (BP).

**Female** (SYNU-F-5802). As in male, except as noted. Measurements: total length 1.11; carapace 0.47 long, 0.39 wide, 0.31 high; abdomen 0.75 long, 0.55 wide, 0.38 high; clypeus 0.07 high; sternum 0.29 long, 0.28 wide. Length of legs: I 1.06 (0.33, 0.12, 0.27, 0.16, 0.18); II 0.96 (0.30, 0.11, 0.22, 0.16, 0.17); III 0.88 (0.28, 0.11, 0.18, 0.15, 0.16); IV 1.16 (0.36, 0.12, 0.28, 0.21, 0.19).

***Carapace*** (Fig. 3A, C, D): ocular area not raised; cephalic part flat. Abdomen (Fig. 3A–C, E): epigynal area with crescent-shaped protrusion (Csp); preanal scutum (Pa) rectangular, with thick posterolateral corners (Plc), ca. 1.5× longer than anal scutum (AS).

***Endogyne*** (Fig. 4A, B): epigynal pit (EP) small; vulval stem (VS) thick; inner vulval plate (IVP) triangular; vulval duct (VD) weakly sclerotized, connected to translucent, saccular seminal receptacle (SR).

##### Distribution.

Indonesia (Sumatra), Thailand (new record) (Fig. 18).

#### Ablemma (Bannia) theppratan

Taxon classification

Animalia

AraneaeTetrablemmidae

Tong & Li sp. nov.

D34AD6A3-CF2F-5E3B-9DF3-4C6671E958D5

https://zoobank.org/F19163B0-E6AF-4866-84FB-16EDCEABD836

**Figure 5.**
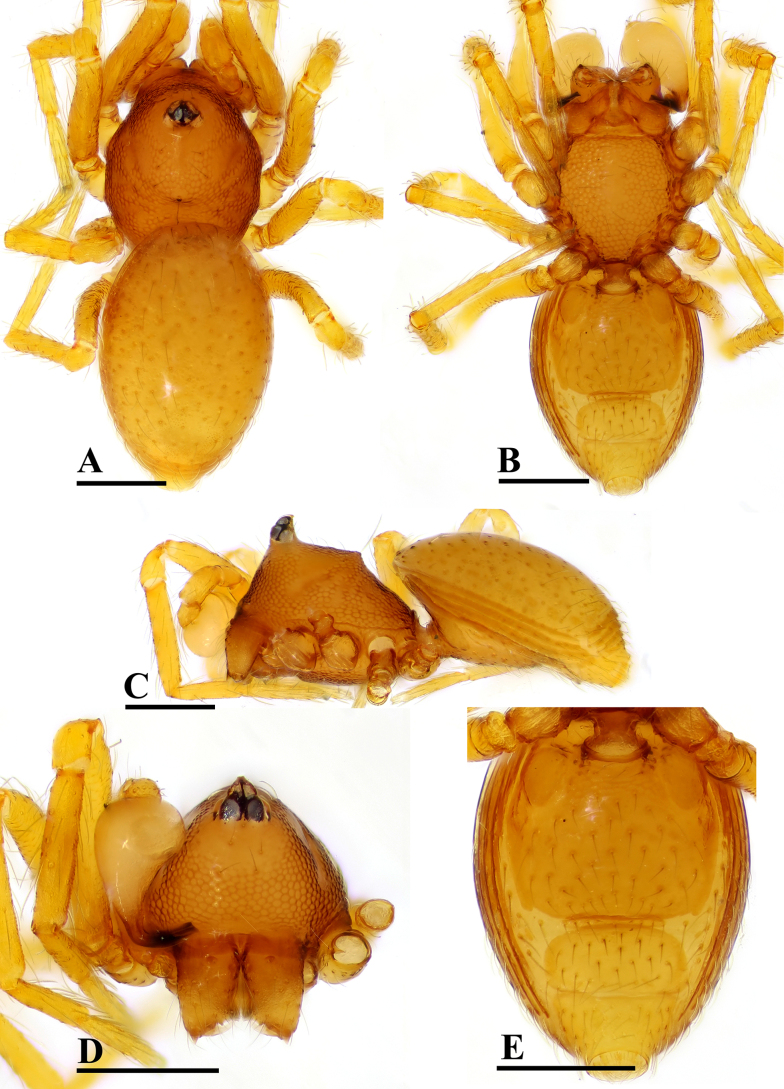
*Ablemma theppratan* sp. nov., male, holotype. **A**. Habitus, dorsal view; **B**. Habitus, ventral view; **C**. Habitus, lateral view; **D**. Prosoma, anterior view; **E**. Abdomen, ventral view. Scale bars: 0.2 mm.

**Figure 6.**
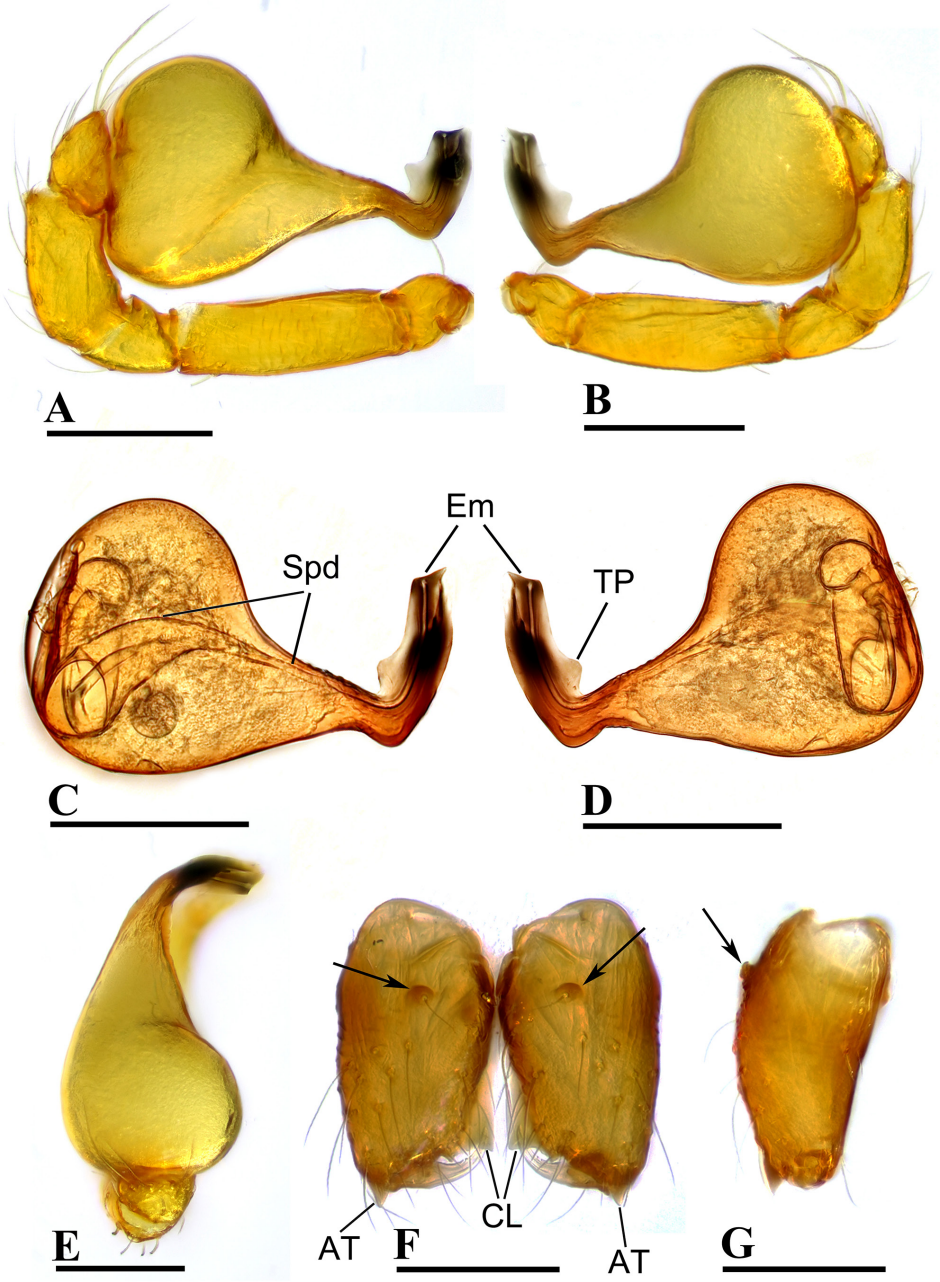
*Ablemma theppratan* sp. nov., male, holotype. **A, C**. Left palp, prolateral view; **B, D**. Left palp, retrolateral view; **E**. Left palp, dorsal view; **F**. Chelicerae, anterior view; **G**. Chelicerae, lateral view; arrows show the outgrowths in (**F**) and (**G**). Abbreviations: AT = anterodistal tooth, CL = cheliceral lamina, Em = embolus, Spd = sperm duct, TP = triangular process. Scale bars: 0.1 mm.

**Figure 7.**
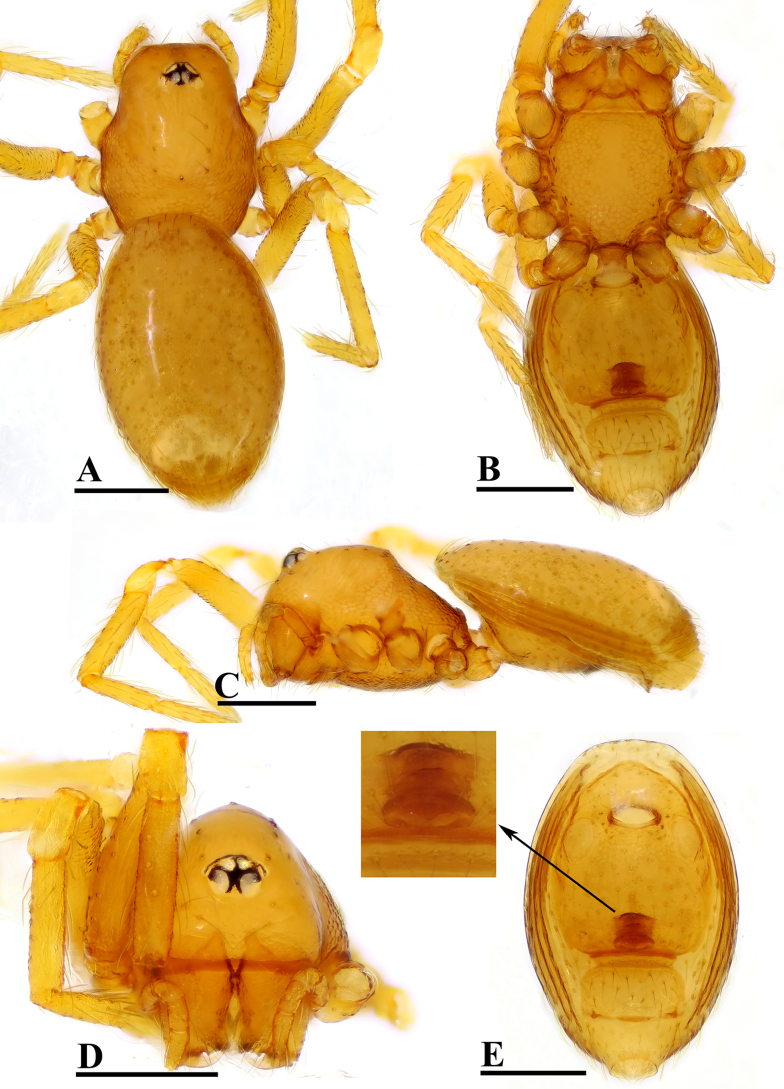
*Ablemma theppratan* sp. nov., female, paratype. **A**. Habitus, dorsal view; **B**. Habitus, ventral view; **C**. Habitus, lateral view; **D**. Prosoma, anterior view; **E**. Abdomen, ventral view, arrow shows detail of genital area. Scale bars: 0.2 mm.

Figs 5, 6, 7, 11A, B

##### Type material.

***Holotype*** Thailand • ♂ (SYNU-F-5565); Krabi, Mueang Dist., Trap Prink Subdist., Thep Pratan Cave (Payanak Cave); 8°10'05.70"N, 98°52'52.40"E, 481 m elev.; 13.X. 2015; H. Zhao et al. leg. ***Paratypes*** Thailand • 2♀ (SYNU-F-5566–5567); same data as holotype.

##### Etymology.

The specific name refers to the type locality and is a noun in apposition.

##### Diagnosis.

The new species is similar to *Ablemma makiling* Lehtinen, 1981 in having subglobular bulb and the belt-shaped embolus, but can be distinguished by the eye tubercle at the anterior part of carapace (vs close to the centre of carapace; cf. Fig. 5C and Lehtinen 1981: fig. 191) and the finger-shaped inner vulval plate (IVP) (vs triangular; cf. Fig. 11B and Lehtinen 1981: fig. 187).

**Figure 8.**
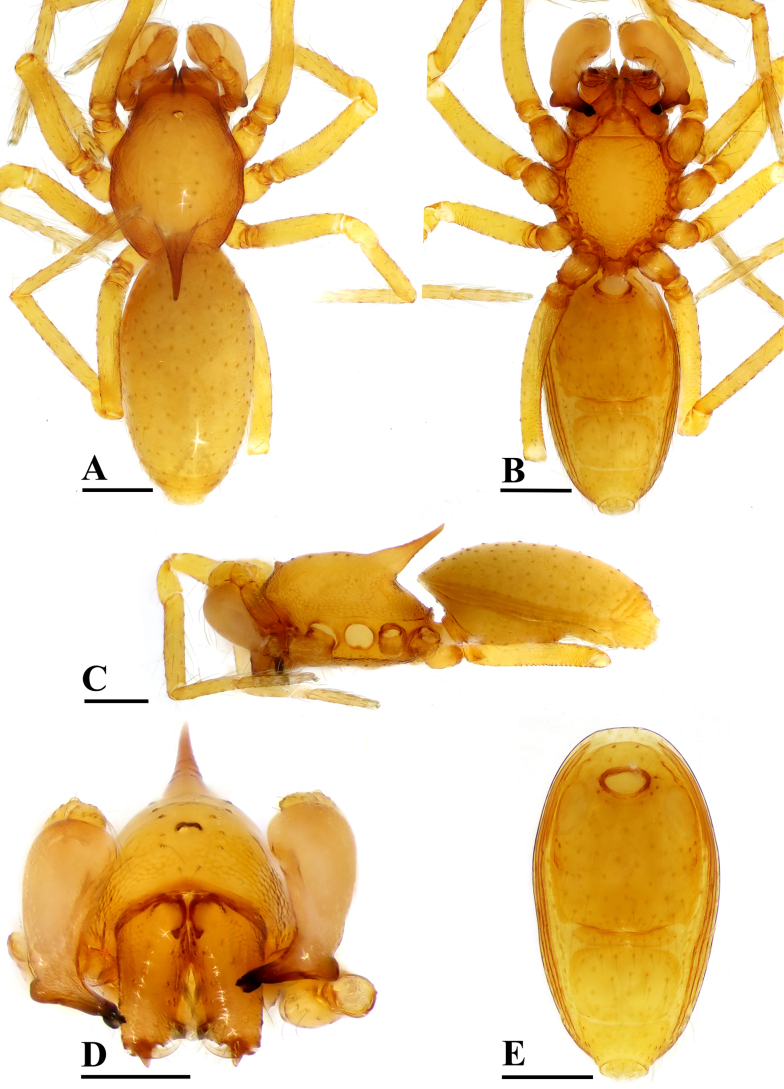
*Ablemma yamae* sp. nov., male, holotype. **A**. Habitus, dorsal view; **B**. Habitus, ventral view; **C**. Habitus, lateral view; **D**. Prosoma, anterior view; **E**. Abdomen, ventral view. Scale bars: 0.2 mm.

**Figure 9.**
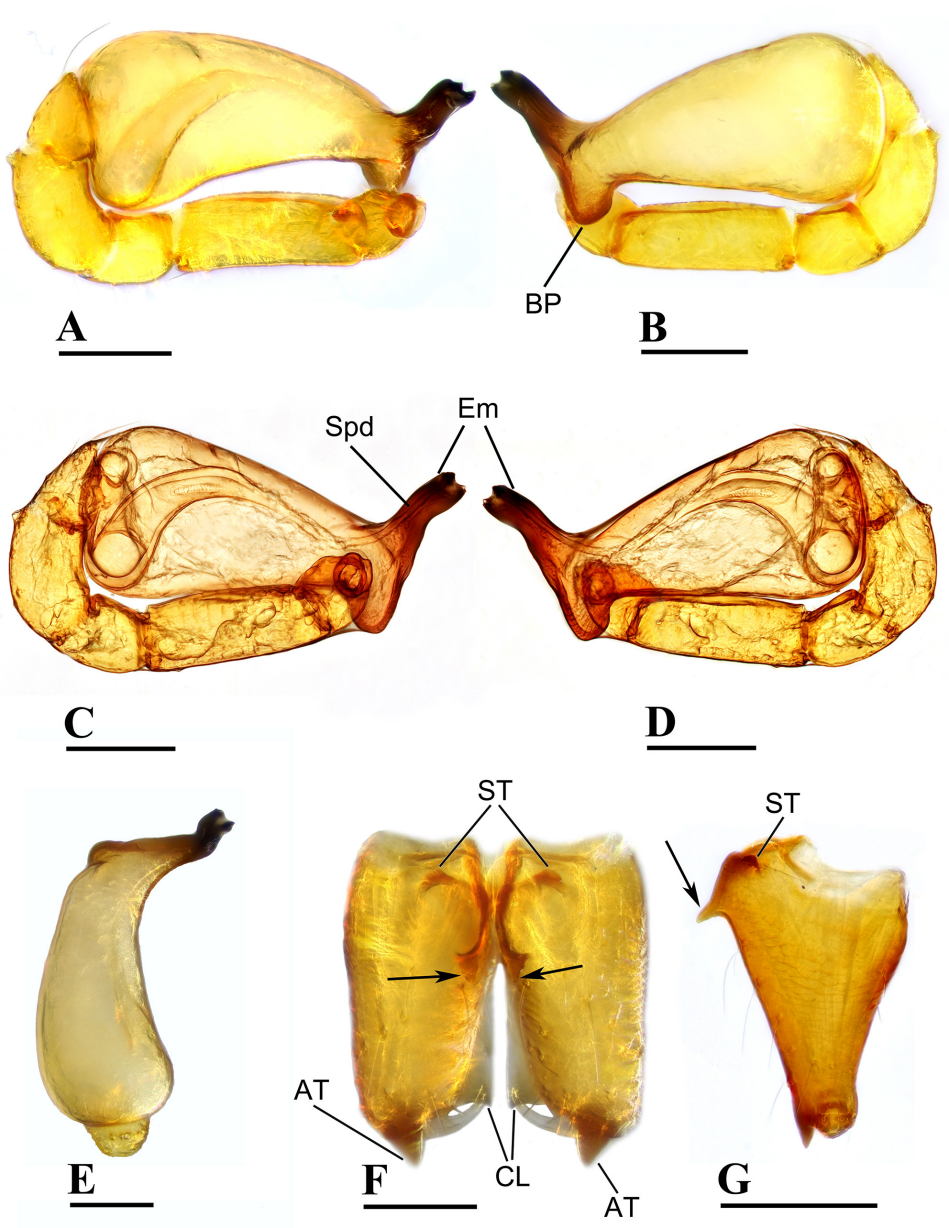
*Ablemma yamae* sp. nov., male, holotype. **A, C**. Left palp, prolateral view; **B, D**. Left palp, retrolateral view; **E**. Left palp, dorsal view; **F**. Chelicerae, anterior view; **G**. Chelicerae, lateral view; arrows show outgrowths in (**F**) and (**G**). Abbreviations: AT = anterodistal tooth, BP = basal process, CL = cheliceral lamina, Em = embolus, Spd = sperm duct, ST = small tubercle. Scale bars: 0.1 mm.

**Figure 10.**
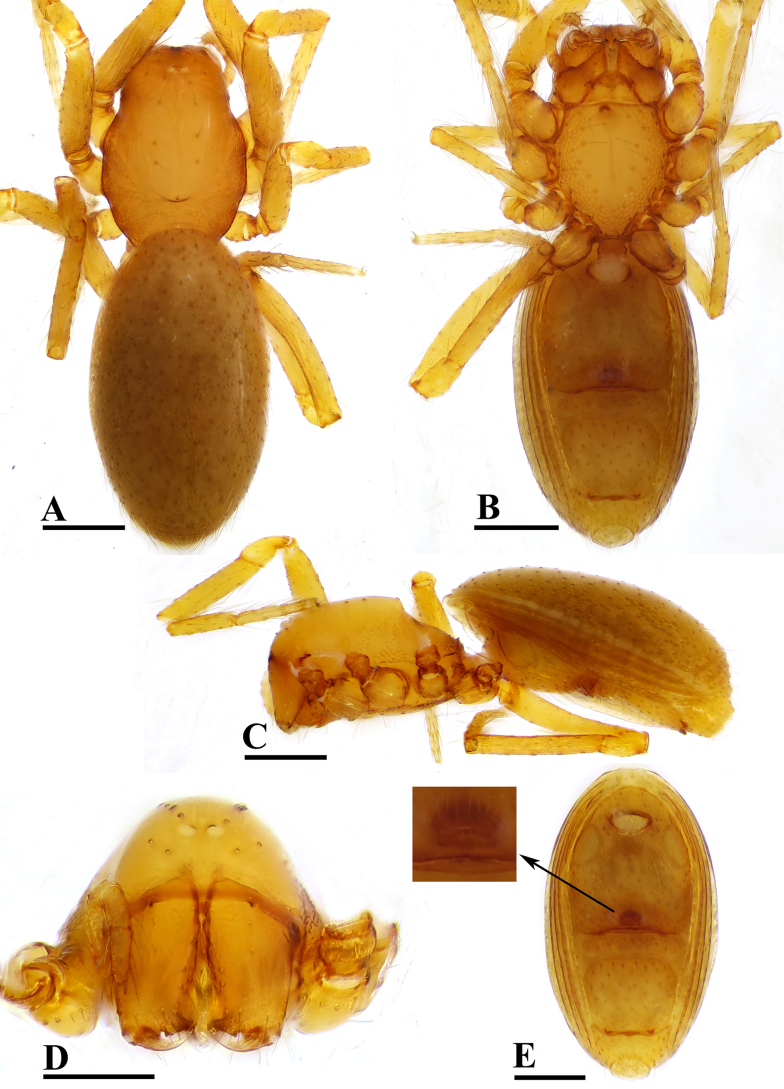
*Ablemma yamae* sp. nov., female, paratype. **A**. Habitus, dorsal view; **B**. Habitus, ventral view; **C**. Habitus, lateral view; **D**. Prosoma, anterior view; **E**. Abdomen, ventral view, arrow shows detail of genital area. Scale bars: 0.2 mm.

**Figure 11.**
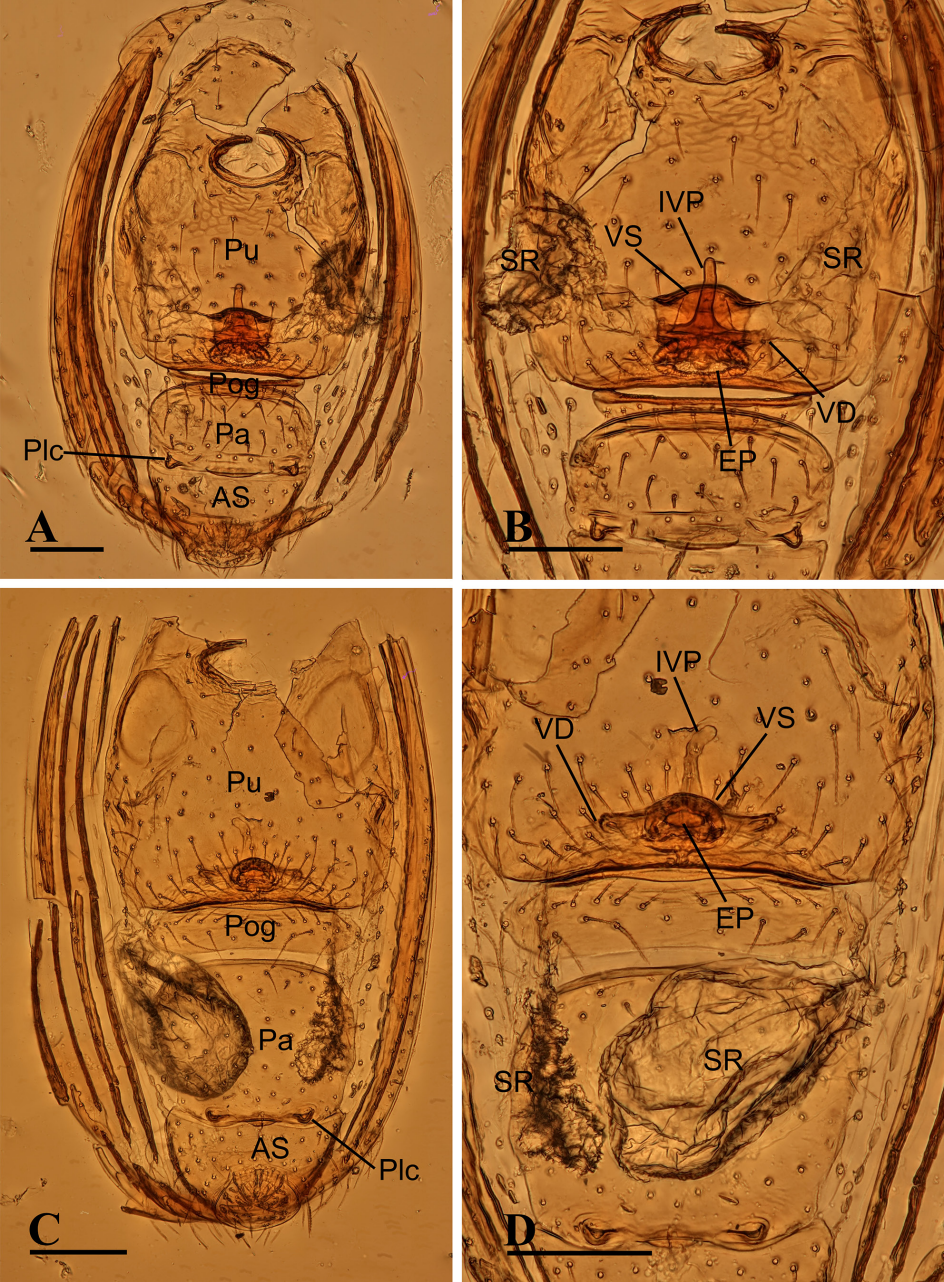
*Ablemma theppratan* sp. nov. (**A, B**); *Ablemma yamae* sp. nov. (**C, D**). **A, C**. Abdomen, ventral view; **B, D**. Endogyne, dorsal view. Abbreviations: AS = anal scutum, EP = epigynal pit, IVP = inner vulval plate, Pa = preanal scutum, Plc = posterolateral corners, Pog = postgenital scutum, Pu = pulmonary scutum, SR = seminal receptacle, VD = vulval duct, VS = vulval stem. Scale bars: 0.1 mm.

##### Description.

**Male** (holotype). Coloration: body yellowish brown; legs yellowish orange. Measurements: total length 0.95; carapace 0.41 long, 0.35 wide, 0.26 high; clypeus 0.28 high; abdomen 0.60 long, 0.39 wide, 0.28 high; sternum 0.28 long, 0.25 wide. Length of legs: I 1.11 (0.36, 0.12, 0.27, 0.17, 0.19); II 0.97 (0.29, 0.11, 0.21, 0.18, 0.18); III 0.87 (0.24, 0.11, 0.18, 0.16, 0.18); IV 1.33 (0.41, 0.12, 0.35, 0.24, 0.21).

***Carapace*** (Fig. 5A, C, D): finely reticulated; 6 eyes, ALE largest, PME smallest, raised on short tubercle; clypeus sloping forward, marginally rounded; cephalic part raised, sloping posteriorly; chelicerae robust, with small outgrowth (arrow in Fig. 6F, G) and an anterodistal tooth (AT), cheliceral lamina (CL) well developed; labium triangular, blunt distally; sternum reticulated except along midline.

***Abdomen*** (Fig. 5A–C, E): dorsal scutum smooth, covered with sparse setae; pulmonary scutum (Pu) with oval book-lung covers; preanal scutum (Pa) large, rectangular, much wider than postgenital scutum (Pog).

***Palp*** (Fig. 6A–E): femur ca. 2.2× longer than patella, 0.8× shorter than bulb; tibia not swollen, ca. 0.8× of femur length; cymbium small, cup-shaped; bulb triangular, narrowing; sperm duct (Spd) broad basally, gradually tapering; embolus (Em) belt-shaped, with a triangular process (TP).

**Female** (paratype, SYNU-F-5566). As in male, except as noted. Measurements: total length 1.02; carapace 0.45 long, 0.33 wide, 0.26 high; clypeus 0.09 high; abdomen 0.64 long, 0.41 wide, 0.29 high; sternum 0.28 long, 0.26 wide. Length of legs: I 1.14 (0.36, 0.11, 0.29, 0.20, 0.18); II 0.98 (0.29, 0.12, 0.22, 0.16, 0.19); III 0.89 (0.26, 0.11, 0.19, 0.16, 017); IV 1.34 (0.43, 0.12, 0.34, 0.26, 0.19).

***Carapace*** (Fig. 7A, C, D): ocular area not raised; cephalic part flat. Abdomen (Fig. 7A–C, E): preanal scutum (Pa) rectangular, with thick posterolateral corners (Plc), as long as anal scutum (AS).

***Endogyne*** (Fig. 11A, B): epigynal pit (EP) large; vulval stem (VS) thick, with strongly sclerotized margins; inner vulval plate (IVP) finger-shaped; vulval duct (VD) weakly sclerotized, connected to translucent, saccular seminal receptacle (SR).

##### Distribution.

Known only from the type locality (Fig. 18).

#### Ablemma (Ablemma) yamae

Taxon classification

Animalia

AraneaeTetrablemmidae

Tong & Li sp. nov.

0B9DE334-9E65-5735-A0EA-2FE31165E556

https://zoobank.org/43A7C797-A0AF-4F06-ADEB-EFE5A5447507

Figs 8, 9, 10, 11C, D

##### Type material.

***Holotype*** Thailand • ♂ (SYNU-F-5589); Tak, Umphang Dist., Ya Mae Cave; 16°02'21.18"N, 98°50'48.72"E, 454 m elev.; 15.XI.2016; H. Zhao et al. leg. ***Paratypes*** Thailand • 2♀ (SYNU-F-5590–5591); same data as holotype.

##### Etymology.

The specific name refers to the type locality and is a noun in apposition.

##### Diagnosis.

The new species is similar to *Ablemma baso* Roewer, 1963, *A. berryi* Shear, 1978 and *A. shimojanai* (Komatsu, 1968) in the large conical projection of carapace, but can be distinguished from *A. baso* by the two reduced eyes (vs two large eyes; cf. Figs 8A, 8D, 10A, 10Dand Lehtinen 1981: fig. 141) and the short boot-shaped embolus (vs very long; cf. Figs 9A–Dand Lehtinen 1981: fig. 138); from *A. berryi* by the two eyes (vs four; cf. Figs 8D, 10Dand Shear 1978: figs 89, 91), the short boot-shaped embolus (vs very long; cf. Figs 9A–Dand Shear 1978: figs 93, 94), and the preanal scutum ca. 4× longer than the postgenital scutum (vs 2.0×; cf. Fig. 10E and Shear 1978: fig. 95); from *A. shimojanai* by the two eyes (vs four eyes; cf. Figs 8D, 10Dand Fu et al. 2022: figs 1G, 2G), the blunt tip of embolus (vs acute tip; cf. Figs 9A–Dand Fu et al. 2022: fig. 3A–F) and the preanal scutum ca. 2.0× longer than anal scutum (vs less than 1.5×; cf. Fig. 10E and Fu et al. 2022: fig. 2H).

##### Description.

**Male** (holotype). Coloration: body yellowish brown; legs yellowish orange. Measurements: total length 1.29; carapace 0.60 long, 0.39 wide, 0.42 high; clypeus 0.11 high; abdomen 0.76 long, 0.40 wide, 0.36 high; sternum 0.35 long, 0.30 wide. Length of legs: I 1.39 (0.43, 0.14, 0.34, 0.22, 0.26); II 1.32 (0.40, 0.15, 0.30, 0.22, 0.25); III 1.17 (0.33, 0.13, 0.27, 0.21, 0.23); IV 1.54 (0.45, 0.15, 0.41, 0.27, 0.26).

***Carapace*** (Fig. 8A, C, D): dorsally smooth, finely reticulated at margin; 2 vestigal eyes; clypeus sloping forward, marginally rounded; cephalic part raised, posterior part with large conical projection, 0.24 high; chelicerae robust, bear­ing one small tubercle (ST); with conical outgrowth (arrow in Fig. 9F, G) and an anterodistal tooth (AT), cheliceral lamina (CL) well developed; labium triangular, blunt distally; sternum reticulated except medial area.

***Abdomen*** (Fig. 8A–C, E): dorsal scutum smooth, covered with sparse setae; pulmonary scutum (Pu) with oval book-lung covers; preanal scutum (Pa) rectangular, nearly same width as postgenital scutum (Pog).

***Palp*** (Fig. 9A–E): femur ca. 2.5× longer than patella, 0.6× shorter than bulb; tibia not swollen, ca. 0.7× of femur length; cymbium small, cup-shaped; bulb pyriform; sperm duct (Spd) broad basally, gradually tapering; embolus (Em) relatively wide, short boot-shaped, distally blunt; basal process (BP) somewhat round.

**Female** (paratype, SYNU-F-5590). As in male, except as noted. Measurements: total length 1.39; carapace 0.58 long, 0.41 wide, 0.31 high; abdomen 0.84 long, 0.50 wide, 0.39 high; clypeus 0.10 high; sternum 0.37 long, 0.30 wide. Length of legs: I 1.36 (0.44, 0.15, 0.32, 0.19, 0.26); II 1.25 (0.39, 0.14, 0.29, 0.18, 0.25); III 1.12 (0.32, 0.14, 0.25, 0.18, 0.23); IV 1.50 (0.45, 0.15, 0.40, 0.25, 0.25).

Cephalic part lacking large conical projection, chelicerae unmodified. Abdomen (Fig. 10A–C, E): preanal scutum (Pa) rectangular, with thick posterolateral corners (Plc) (Fig. 10E), ca. 2.0× longer than anal scutum (AS).

***Endogyne*** (Fig. 11C, D): epigynal pit (EP) small; inner vulval plate (IVP) finger-shaped; vulval duct (VD) weakly sclerotized, connected to translucent, saccular seminal receptacle (SR).

##### Distribution.

Known only from the type locality (Fig. 18).

### Subfamily Tetrablemminae Lehtinen, 1981

#### Genus Shearella

Taxon classification

Animalia

AraneaeTetrablemmidae

Lehtinen, 1981

8AE2C6CB-19DB-5C45-9844-94EDD5370C73

##### Type species.

*Shearella lilawati* Lehtinen, 1981 from Ceylon.

##### Diagnosis.

See Lehtinen (1981).

##### Composition.

Seven species, including two described here.

##### Distribution.

China (1 species), India (1), Madagascar (1), Sri Lanka (2) and Thailand (2).

#### Shearella khaoplu

Taxon classification

Animalia

AraneaeTetrablemmidae

Tong & Li sp. nov.

D0D7E67F-7FE9-5D23-BBF8-17744A786E5E

https://zoobank.org/369533F6-C43B-4F80-9C1E-CF62589E3B39

Figs 12, 13

##### Type material.

***Holotype*** Thailand • ♂ (SYNU-F-5532); Nakhon Srithammarat, Thung Song Dist., Khao Ro Subdist., Khao Plu Cave; 8°01'22.00"N, 99°34'35.30"E, 55 m elev.; 13.X.2015; H. Zhao et al. leg.

**Figure 12.**
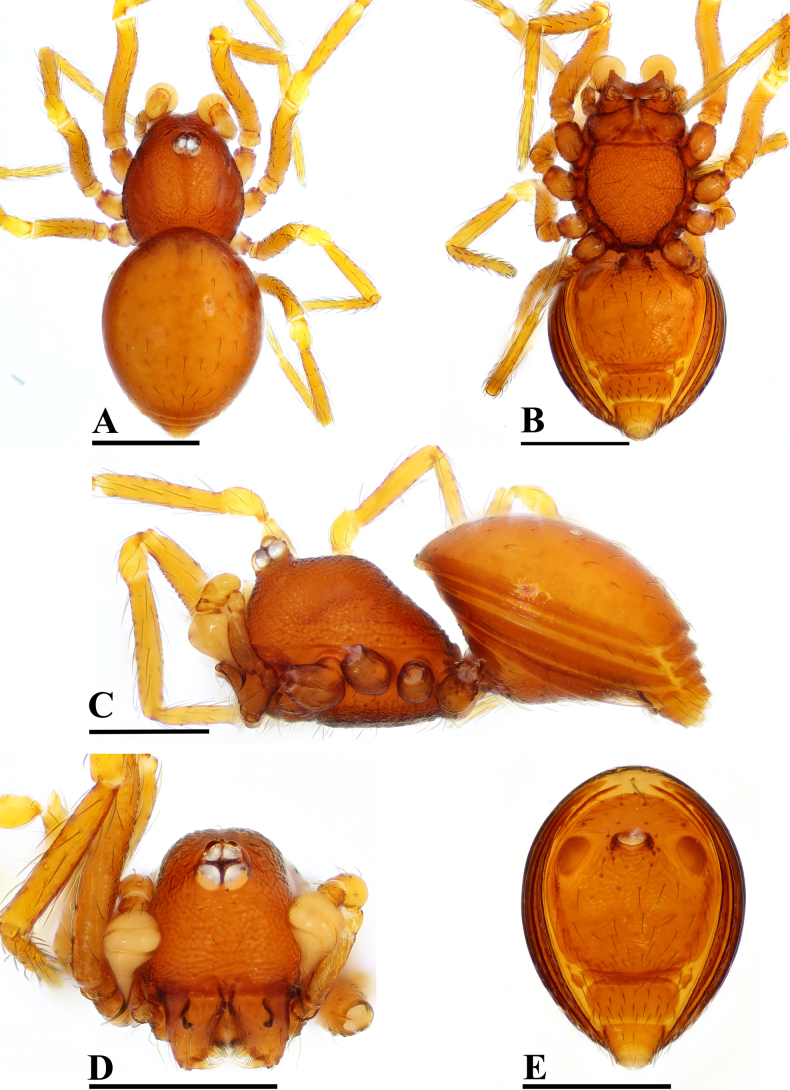
*Shearella khaoplu* sp. nov., male, holotype. **A**. Habitus, dorsal view; **B**. Habitus, ventral view; **C**. Habitus, lateral view; **D**. Prosoma, anterior view; **E**. Abdomen, ventral view. Scale bars: 0.4 mm.

**Figure 13.**
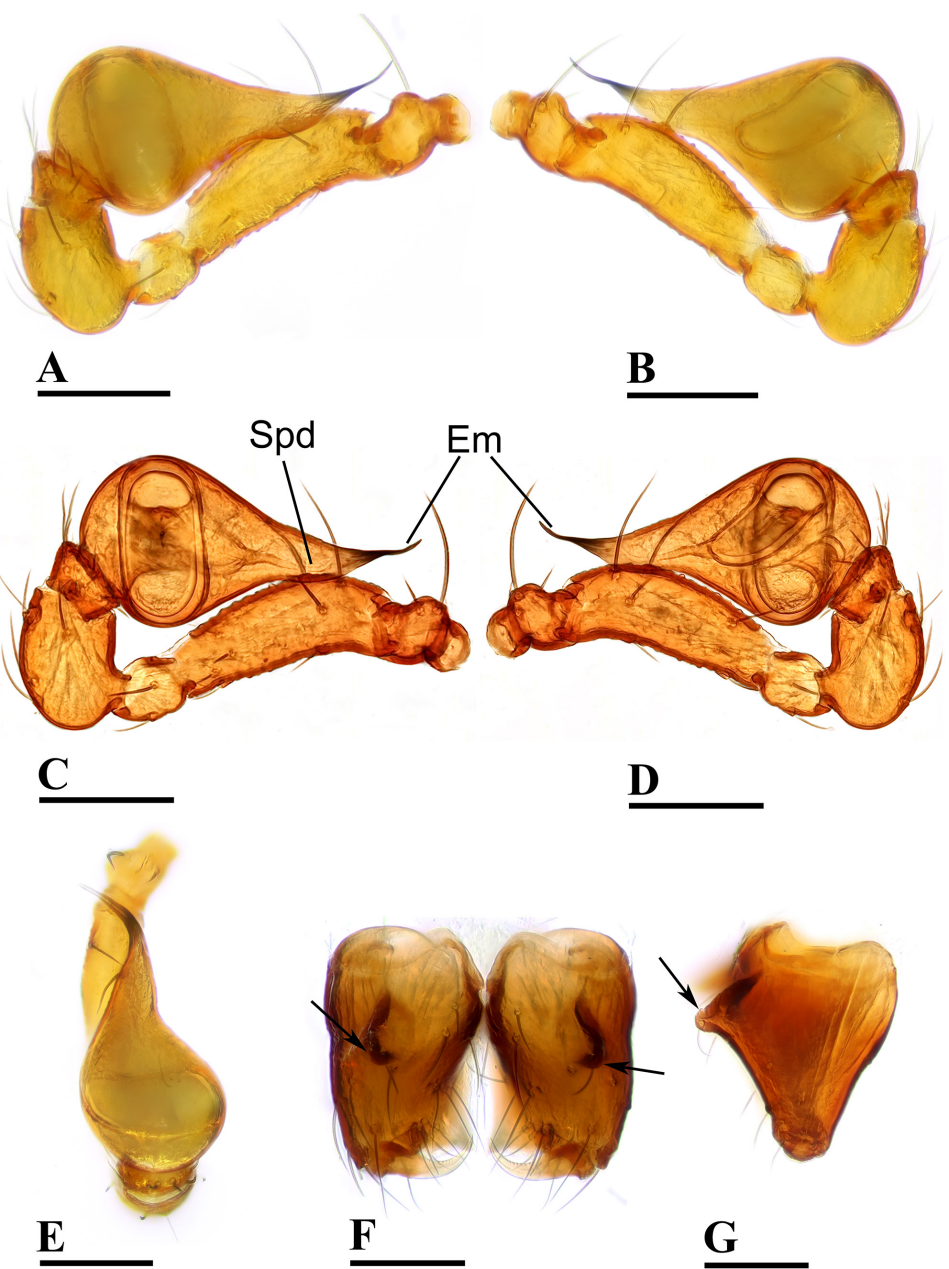
*Shearella khaoplu* sp. nov., male, holotype. **A, C**. Left palp, prolateral view; **B, D**. Left palp, retrolateral view; **E**. Left palp, dorsal view; **F**. Chelicerae, anterior view; **G**. Chelicerae, lateral view; arrows show conical horn in (**F**) and (**G**). Abbreviations: Em = embolus, Spd = sperm duct. Scale bars: 0.1 mm.

##### Etymology.

The specific name refers to the type locality and is a noun in apposition.

##### Diagnosis.

The new species is similar to *Shearella lilawati* Lehtinen, 1981 in the short eye tubercle, but can be distinguished by the long, distally narrow bulb (vs oval; cf. Fig. 13A–D and Lehtinen 1981: fig. 214) and the blunt tip of cheliceral horn (vs acute tip; cf. Fig. 13F, G and Lehtinen 1981: fig. 202).

##### Description.

**Male** (holotype). Coloration: body reddish-brown; legs yellowish brown. Measurements: total length 1.22; carapace 0.53 long, 0.46 wide, 0.43 high; abdomen 0.82 long, 0.61 wide, 0.54 high; clypeus 0.21 high; sternum 0.39 long, 0.37 wide. Length of legs: I 1.51 (0.50, 0.14, 0.38, 0.25, 0.24); II 1.29 (0.42, 0.13, 0.32, 0.20, 0.22); III 1.23 (0.39, 0.13, 0.27, 0.22, 0.22); IV 1.60 (0.50, 0.14, 0.41, 0.29, 0.26).

***Carapace*** (Fig. 12A, C, D): reticulated; 6 eyes, ALE largest, PME smallest, raised on short tubercle; clypeus sloping forward, with sparse setae; chelicerae frontally with basally wide, short horn (arrow in Fig. 13F, G), cheliceral lamina (CL) developed; endites basally wide, distally narrow, labium triangular, distally blunt; sternum reticulated.

***Abdomen*** (Fig. 12A–C, E): dorsal scutum surface smooth, posteriorly truncated; ventral scutum smooth; lateral scutum I long, and exceeding beyond the posterior margin of preanal scutum (Pa); postgenital scutum (Pog) straight; preanal scutum (Pa) rectangular, ca. 3.0× longer than the postgenital scutum (Pog).

***Palp*** (Fig. 13A–E): femora 3.0× longer than patella, 0.7× shorter than bulb, with several long setae; tibia large, swollen, ca. 1.2× wider than femur, 0.7× shorter than femur; cymbium short, triangular from lateral view; bulb long (length/width ca. 1.6), distally narrow, gradually tapering; embolus (Em) thin, straight; sperm duct (Spd) coiled into 2 loops, gradually twisting to narrow, and open at embolic tip.

**Female**. Unknown.

##### Distribution.

Known only from the type locality (Fig. 18).

#### Shearella thamphothisat

Taxon classification

Animalia

AraneaeTetrablemmidae

Tong & Li sp. nov.

50AC6BDB-7A8C-5002-8519-70A738CFF339

https://zoobank.org/B22809C1-DB2C-4948-91DB-AC3F9889D710

Figs 14, 15

##### Type material.

***Holotype*** Thailand • ♂ (SYNU-F-5433); Saraburi, Kaeng Koi Dist., Tap Khwang Subdist., Nam pu Vill., Tham Phothisat Cave; 14°34'27.90"N, 101°08'52.02"E, 230 m elev.; 20.X.2014; H. Zhao et al. leg.

**Figure 14.**
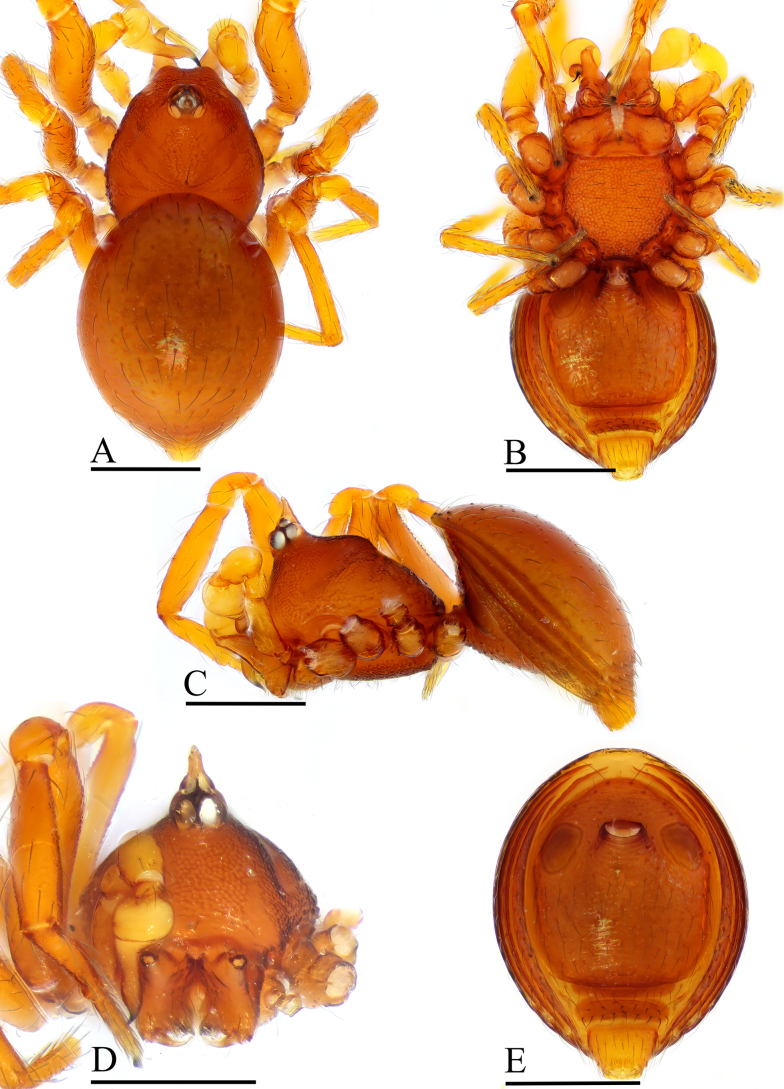
*Shearella thamphothisat* sp. nov., male, holotype. **A**. Habitus, dorsal view; **B**. Habitus, ventral view; **C**. Habitus, lateral view; **D**. Prosoma, anterior view; **E**. Abdomen, ventral view. Scale bars: 0.4 mm.

**Figure 15.**
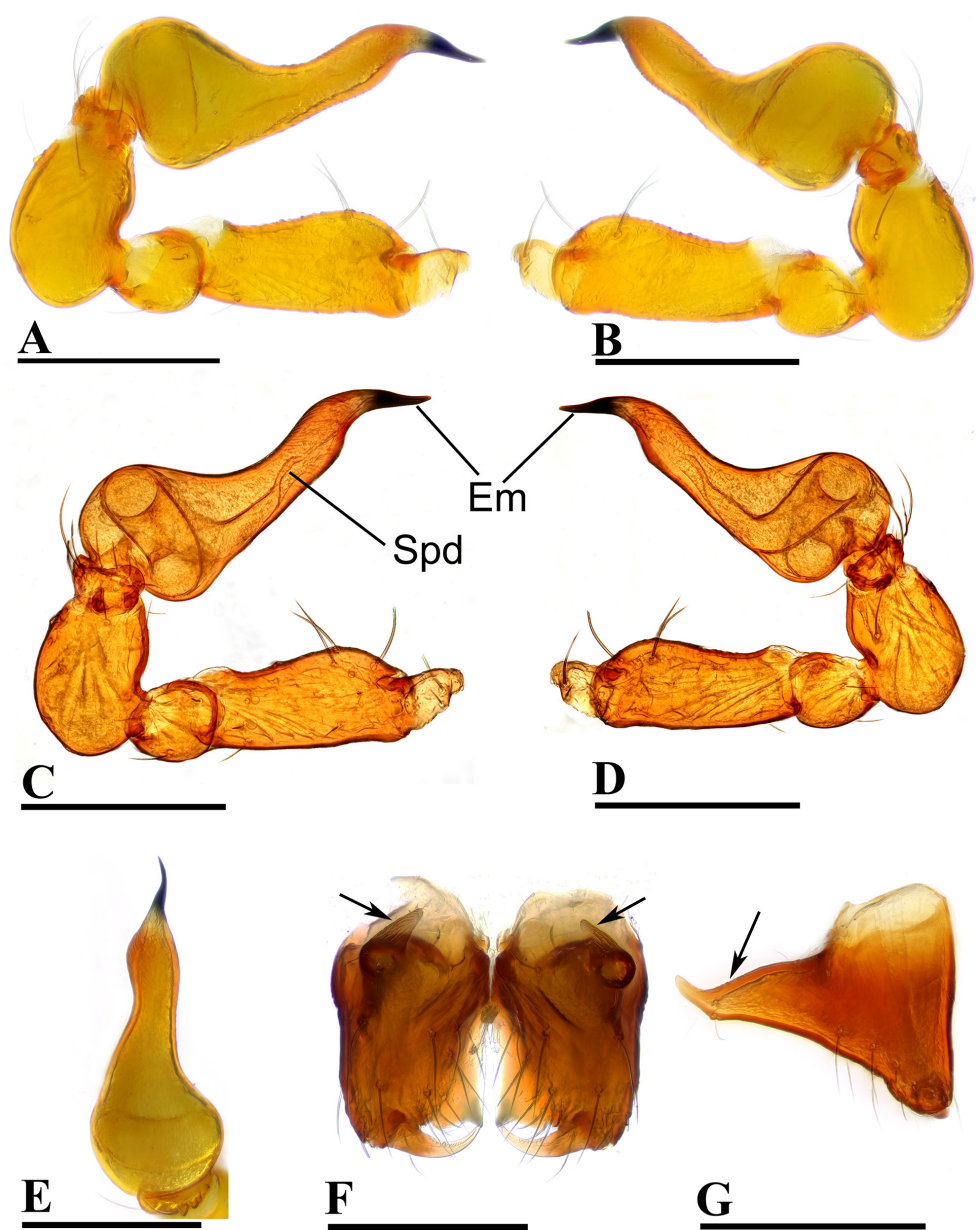
*Shearella thamphothisat* sp. nov., male, holotype. **A, C**. Left palp, prolateral view; **B, D**. Left palp, retrolateral view; **E**. Left palp, dorsal view; **F**. Chelicerae, anterior view; **G**. Chelicerae, lateral view; arrows show large conical horn in (**F**) and (**G**). Abbreviations: Em = embolus, Spd = sperm duct. Scale bars: 0.2 mm.

##### Etymology.

The specific name refers to the type locality and is a noun in apposition.

##### Diagnosis.

The new species is similar to *Shearella sanya* Lin & Li, 2010 in the acute eye tubercle, but can be distinguished by the very long bulb (vs pear-shaped; cf. Fig. 15A–D and Lin and Li 2010: figs 23, 24) and the pointed cheliceral horn (vs dichotomous; cf. Fig. 15F, G and Lin and Li 2010: figs 19, 20).

##### Description.

**Male** (holotype). Coloration: body reddish-brown; legs yellowish brown. Measurements: total length 1.59; carapace 0.67 long, 0.58 wide, 0.46 high; abdomen 0.99 long, 0.76 wide, 0.46 high; clypeus 0.28 high; sternum 0.38 long, 0.42 wide. Length of legs: I 1.71 (0.55, 0.18, 0.45, 0.30, 0.23); II 1.65 (0.52, 0.18, 0.45, 0.28, 0.22); III 1.53 (0.50, 0.17, 0.38, 0.26, 0.22); IV 1.87 (0.61, 0.20, 0.47, 0.33, 0.26).

***Carapace*** (Fig. 14A, C, D): reticulated; 6 eyes, ALE largest, PME smallest, raised on long, conical tubercle; clypeus sloping forward, with sparse setae; chelicerae frontally with basally wide, pointed horn (arrow in Fig. 15F, G), cheliceral lamina (CL) developed; endites basally wide, distally narrow, labium triangular, distally blunt; sternum reticulated.

***Abdomen*** (Fig. 14A–C, E): dorsal scutum surface smooth, posteriorly truncated; ventral scutum smooth; lateral scutum I long, and exceeding beyond the posterior margin of preanal scutum (Pa); postgenital scutum (Pog) straight; preanal scutum (Pa) rectangular, ca. 2.0× longer than the postgenital scutum (Pog).

***Palp*** (Fig. 15A–E): femora 2.3× longer than patella, 0.7× shorter than bulb; tibia large, swollen, ca. 1.2× wider than femur, 0.8× shorter than femur; cymbium short, triangular from lateral view; bulb very long (length/width ca. 1.8), basally subglobular, gradually tapering; embolus (Em) tubular; sperm duct (Spd) coiled into 1 loop, gradually twisting to narrow, and open at embolic tip.

**Female**. Unknown.

##### Distribution.

Known only from the type locality (Fig. 18).

#### Genus Tetrablemma

Taxon classification

Animalia

AraneaeTetrablemmidae

O. Pickard-Cambridge, 1873

D82DC053-9542-5293-95B3-98220A5FCF34

##### Type species.

*Tetrablemma medioculatum* O. Pickard-Cambridge, 1873 from Sri Lanka.

##### Diagnosis.

See Lehtinen (1981).

##### Composition.

Thirty-three species, including one described here in three subgenera, subgenera *Kumaonia* Lentinen, 1981, *Indonops* Tikader, 1975 and *Tetrablemma* O. Pickard-Cambridge, 1873.

##### Distribution.

Angola (2 species), Australia (3), Cambodia (2), China (5), Colombia (1), India (5), Indonesia (3), Laos (1), Micronesia (1), Myanmar (1), Nepal (1), Samoa (1), Seychelles (1), Sri Lanka (1), St. Helena (1), Thailand (1), Trinidad (1), Venezuela (1) and Vietnam (1).

#### Tetrablemma (Kumaonia) lorkor

Taxon classification

Animalia

AraneaeTetrablemmidae

Tong & Li sp. nov.

481BA83C-0AE9-5516-A053-9052DB001C17

https://zoobank.org/05B62CB3-A704-4774-942F-4D41BBC4F907

Figs 16, 17

##### Type material.

***Holotype*** Thailand • ♂ (SYNU-F-5996); Rattalung, Khao Chaison Dist., Lor Kor Cave; 7°26'53.90"N, 100°07'31.20"E, 14 m elev.; 26.X.2015; H. Zhao et al. leg.

**Figure 16.**
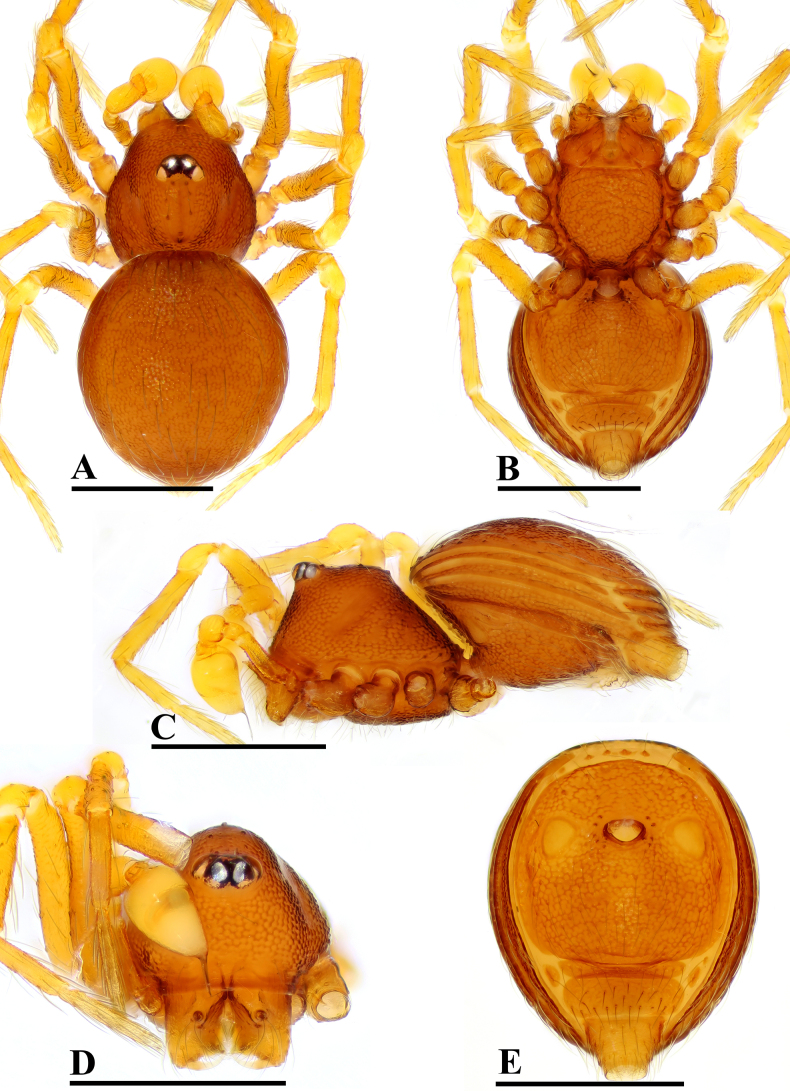
*Tetrablemma lorkor* sp. nov., male, holotype. **A**. Habitus, dorsal view; **B**. Habitus, ventral view; **C**. Habitus, lateral view; **D**. Prosoma, anterior view; **E**. Abdomen, ventral view. Scale bars: 0.4 mm.

**Figure 17.**
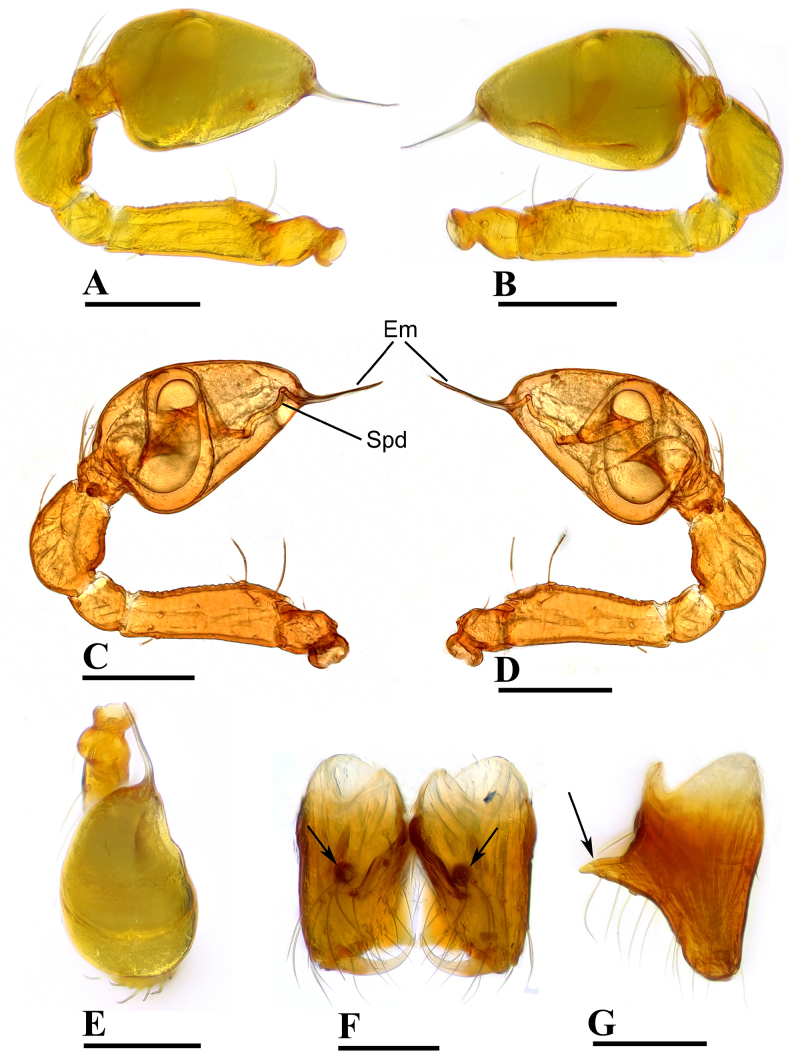
*Tetrablemma lorkor* sp. nov., male, holotype. **A, C**. Left palp, prolateral view; **B, D**. Left palp, retrolateral view; **E**. Left palp, dorsal view; **F**. Chelicerae, anterior view; **G**. Chelicerae, lateral view; arrows show large conical horn in (**F**) and (**G**). Abbreviations: Em = embolus, Spd = sperm duct. Scale bars: 0.1 mm.

**Figure 18.**
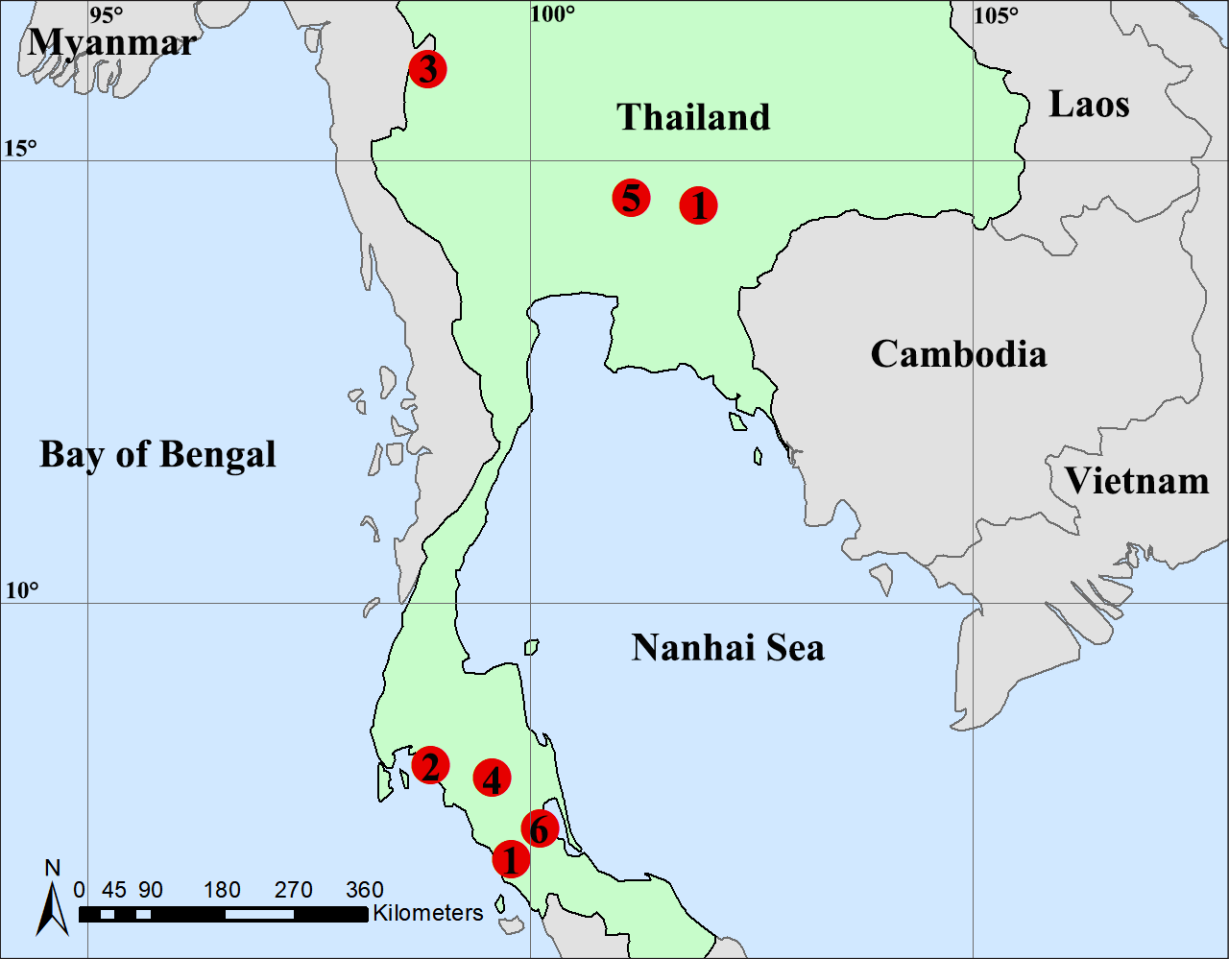
Distribution records of six tetrablemmids species from Thailand: *Ablemma erna* Lehtinen, 1981 (1); *A. theppratan* sp. nov. (2); *A. yamae* sp. nov. (3); *Shearella khaoplu* sp. nov. (4); *S. thamphothisat* sp. nov. (5); *Tetrablemma lorkor* sp. nov. (6).

##### Etymology.

The specific name refers to the type locality and is a noun in apposition.

##### Diagnosis.

The new species is similar to *Tetrablemma loebli* Bourne, 1980 in the oval bulb and thin embolus, but can be distinguished by the PLE separated by more than twice their diameter (vs less than one diameter; cf. Fig. 16A and Lehtinen 1981: fig. 219), the irregularly reticulated sternum (vs reticulated except medially; cf. Fig. 16B and Lehtinen 1981: fig. 220), and the short conical cheliceral horns (vs very long, falciform; cf. Fig. 17G and Lehtinen 1981: fig. 221).

##### Description.

**Male** (holotype). Coloration: body orange; legs yellowish orange. Measurements: total length 1.09; carapace 0.47 long, 0.43 wide, 0.32 high; abdomen 0.70 long, 0.58 wide, 0.37 high; clypeus 0.18 high; sternum 0.28 long, 0.31 wide. Length of legs: I 1.18 (0.42, 0.12, 0.25, 0.18, 0.21); II 1.07 (0.33, 0.12, 0.24, 0.18, 0.20); III 0.95 (0.29, 0.11, 0.22, 0.16, 0.17); IV 1.27 (0.41, 0.12, 0.30, 0.21, 0.23).

***Carapace*** (Fig. 16A, C): finely reticulated, ocular area slightly raised; 4 eyes, ALE almost touching, ALE> PLE, PLE separated by more than twice their diameter; clypeus sloping forward, with sparse setae, marginally round; cheliceral horns short, conical (arrows in Fig. 17F, G), cheliceral lamina (CL) developed; endites basally wide, distally narrow, labium triangular, distally blunt; sternum irregularly reticulated.

***Abdomen*** (Fig. 16A–C, E): dorsal scutum finely reticulated, posteriorly truncated; ventral scutum reticulated; lateral scutum I long, and exceeding beyond the posterior margin of preanal scutum (Pa); postgenital scutum (Pog) straight; preanal scutum (Pa) rectangular, ca. 3.0× longer than the postgenital scutum (Pog).

***Palp*** (Fig. 17A–E): femoral cuticle granulated, ca. 3.0× longer than patella, 0.8× shorter than bulb; tibia large, swollen, ca. 1.5× wider than femur, 0.7× shorter than femur; cymbium short, triangular from lateral view; bulb oval; embolus (Em) thin, straight; sperm duct (Spd) coiled into 2 loops, abruptly twisting to narrow, and open at embolic tip.

**Female**. Unknown.

##### Distribution.

Known only from the type locality (Fig. 18).

## Supplementary Material

XML Treatment for Ablemma

XML Treatment for Ablemma (Bannia) erna

XML Treatment for Ablemma (Bannia) theppratan

XML Treatment for Ablemma (Ablemma) yamae

XML Treatment for Shearella

XML Treatment for Shearella khaoplu

XML Treatment for Shearella thamphothisat

XML Treatment for Tetrablemma

XML Treatment for Tetrablemma (Kumaonia) lorkor

## Additional information

### Artificial Intelligence (AI) use

The authors used Doubao for language refinement in the manuscript.

### Author ORCIDs

S. Shi https://orcid.org/0009-0003-6482-9926

D. Bian https://orcid.org/0000-0002-9324-8026

Y. Tong https://orcid.org/0000-0002-4348-7029

S. Li https://orcid.org/0000-0002-3290-5416

### Data availability

All of the data that support the findings of this study are available in the main text.
